# Preoperative levels of biomarkers of coagulation to predict massive bleeding after surgical repair for acute type A aortic dissection: a retrospective cohort study

**DOI:** 10.1186/s13019-026-04692-8

**Published:** 2026-10-12

**Authors:** Emilia Norén, Jacob Ede, Mårten Larsson, Shahab Nozohoor, Johan Sjögren, Karl Teurneau-Hermansson

**Affiliations:** Department of Cardiothoracic Surgery, Skåne University Hospital, Lund University, 221 85 Lund, SE Sweden

**Keywords:** Aortic dissection, Dissection, Ascending Aorta, Postoperative hemorrhage, Retrospective studies, Predictive value of tests

## Abstract

**Background:**

Acute type A aortic dissection is a highly emergent condition with a high mortality rate. The condition poses an imminent risk of serious complications such as coagulopathy with massive bleeding. This increases the risk of mortality even after surgical repair. Procoagulant drugs are routinely administered but some patients still suffer postoperative massive bleeding that can be fatal. The study aimed to investigate whether massive bleeding after surgical treatment for type A aortic dissection can be predicted preoperatively through routine coagulation tests such as APTT, PT (INR), fibrinogen, D-dimer, and platelets.

**Methods:**

The study was a retrospective, single-center, cohort study performed at a tertiary cardiac surgery unit. A total of 474 patients who underwent surgery for type A aortic dissection at our department between January 2005 and May 2023 were included in the study. Of these, 87 patients had massive postoperative bleeding after surgery for type A aortic dissection and were compared to 387 patients who did not have massive postoperative bleeding. Uni- and multivariable analyses were performed using logistic regression. The multivariable model included predictors of massive bleeding. Bleeding-related routine lab variables were then forced into the model.

**Results:**

Patients with massive bleeding (18%) had higher preoperative activated partial thromboplastin time (APTT) (33 s vs 29 s, *p*<0.01), and creatinine (97 µmol/L vs 87 µmol/L, *p*<0.01), as well as lower preoperative platelet counts (172 × 10^9^/L vs 187 × 10^9^/L, *p*=0.03) compared to patients without massive bleeding. Patients with massive bleeding also had longer duration of cardiopulmonary bypass (CPB) (221 min vs 170 min, *p*<0.01) and suffered more postoperative complications. Multivariable logistic regression identified preoperative APTT per mmol increment (OR 1.04 (95% CI 1.01–1.07, *p*=0.01), preoperative APTT > 34.5 s (OR 2.95 (95% CI 1.60–5.47), *p*<0.01), preoperative creatinine > 82.5 µmol/L (OR 2.16 (95% CI 1.15–4.06), *p*=0.02), hypertension (OR 1.83 (95% CI 1.01–3.32), *p*<0.05) and CPB time per 10 min increment (OR 1.09 (95% CI 1.05–1.13, *p*<0.01)) as independent predictors of massive bleeding.

**Conclusion:**

Preoperative APTT exceeding 34.5 s was independently associated with massive bleeding after type A aortic dissection repair and may, pending prospective validation, help identify patients who could potentially benefit from earlier hemostatic intervention.

**Supplementary Information:**

The online version contains supplementary material available at https://doi.org/10.1186/s13019-026-04692-8.

## Background

Acute type A aortic dissection (ATAAD) is a highly emergent condition with an incidence of 2.5–6 cases per 100,000 patient years [1, 2] and a prehospital mortality rate of up to 47% [1, 3]. Even after surgical repair, the 30-day mortality is about 17% [4]. There is also an imminent risk of serious complications such as neurological injury, kidney failure, and coagulopathy with massive bleeding, the latter increasing the risk of mortality after surgical repair [5, 6].

The coagulopathy of ATAAD can mainly be attributed to the false lumen, with large surfaces expressing tissue factor and other substances that activate the coagulation system, including systemic fibrinolysis and thrombin activation. This process can ultimately lead to disseminated intravascular coagulation (DIC), dramatically increasing the risk of fatal bleeding [7–11]. In addition, factors associated with the surgical procedure including cardiopulmonary bypass (CPB) and hypothermia may also aggravate the hemostatic changes [12, 13]. Cardiopulmonary bypass risks worsening the coagulopathy as it decreases concentration of coagulation factors such as fibrinogen and prothrombin. It also lowers platelet concentration and impairs platelet function [13–15].

While preoperative coagulation markers such as APTT and platelet count have been studied as predictors of mortality and prognosis in ATAAD [16], their value for predicting massive postoperative bleeding specifically has received little attention. We hypothesized that the coagulopathy associated with ATAAD might be reflected in preoperative parameters related to coagulation pathways, and that the levels of these biomarkers could predict the risk of massive postoperative bleeding. It would be beneficial to preoperatively identify patients at high risk for massive bleeding as it could enable earlier hemostatic intervention for patients who may benefit from a more aggressive procoagulant therapy.

## Methods

### Aim

The aim of this study was to investigate whether massive bleeding after ATAAD surgery could be predicted through standard preoperative coagulation-related laboratory tests such as APTT, PT (INR), fibrinogen, D-dimer, or platelets.

### Study design

This was a retrospective, single center, observational, cohort study performed at a tertiary referral center with a catchment area of 1.9 million inhabitants. All patients who underwent and survived surgical repair for ATAAD at the department of Cardiothoracic Surgery at Skåne University Hospital in Lund, Sweden, between January 2005 and May 2023 were assessed for inclusion. All patients with complete data on bleeding, transfusions, reoperations, and post-operative death were included.

Data was collected from our departmental surgical database, and additional data on bleeding events, transfusions, and laboratory data (APTT, PT (INR), platelet count, fibrinogen, and D-dimer) were retrospectively collected from the patients’ charts. If patients had multiple preoperative laboratory values recorded, the value closest to the surgical repair was selected.

The primary outcome measure of this study was massive bleeding. Secondary outcome measures were 30-day mortality, 90-day mortality, and postoperative complications. Massive bleeding was defined in accordance with the BART-criteria [17], and patients who met one or more of the following criteria were considered to have massive bleeding: postoperative bleeding through chest tubes exceeding 1500 ml over any 8 h period; reoperation for bleeding or cardiac tamponade within 24 h of surgery; transfusion of more than 10 U of red blood cells within 24 h after surgery; or death from hemorrhage within 30 days. Patients who did not meet any of the criteria were assigned to the control group.

### Definitions

In this study, ATAAD was defined as a Stanford type A aortic dissection with a symptom duration of less than 14 days. Hypotensive shock was defined as preoperative systolic blood pressure below 90 mmHg. Malperfusion was defined as end-organ ischemia identified either clinically or by objective testing (imaging, biochemistry, or electrocardiography), and was recorded per organ system. These categories map to the malperfusion (M) component of the TEM classification: coronary malperfusion (M1), supra-aortic malperfusion (M2; cerebral and innominate territories), and visceral/renal or lower-extremity malperfusion (M3; renal, gastrointestinal, and ileofemoral territories) [18]. As spinal malperfusion is not explicitly included in the original TEM classification, it was classified as M3 in this study. Patients without malperfusion were classified as M0. The clinical-versus-radiological (+ / −) qualifier of the TEM classification was not applied, as the dataset does not record at the individual patient level whether malperfusion was clinically manifest or detected by objective testing. For analysis, preoperative APTT, platelet count, and creatinine were dichotomized at thresholds derived by ROC analysis and Youden's index: APTT >34.5 s, platelet count <166.5 × 10⁹/L, and creatinine >82.5 µmol/L. Postoperative complications recorded as secondary outcomes included stroke and cardiac tamponade, coma, atrial fibrillation, myocardial infarction, superficial and deep sternal wound infection, and sepsis. Stroke: focal neurological deficit persisting for at least 24 h, with, or without, radiological confirmation. Tamponade: pericardial effusion with hemodynamic compromise (systolic blood pressure < 90 mm Hg without other reasonable cause). Coma: unconsciousness > 48 h after termination of sedation. Atrial fibrillation: atrial fibrillation or atrial flutter lasting > 30 s on telemetry or ECG. Myocardial infarction: CKMB > 70 μg/L. Superficial wound infection: infection of the sternotomy wound requiring antibiotic treatment. Deep sternal wound infection: infection at the site requiring antibiotic treatment and vacuum assisted closure-therapy. Sepsis: clinical signs of infection and positive blood culture.

### Surgical techniques

The surgical principles of ATAAD-repair at the Department of Cardiothoracic Surgery, Skåne University Hospital, Lund, have previously been described [19]. All procedures were performed with general anesthesia, median sternotomy, cardiopulmonary bypass, and intermittent cold blood cardioplegia. Specific details in the surgical technique and bleeding management were left to the discretion of each responsible surgeon. However, 4 g of tranexamic acid was routinely administered, and at the end of CPB, 4 units of platelets were transfused. Since 2008, 2 g of fibrinogen concentrate also has routinely been administered at the end of CPB. In addition, since 2015, a standardized bleeding management protocol was routinely used, where coagulation was measured through blood analysis (B-haemoglobin, platelets, P-fibrinogen, P-PT (INR), and P-APTT) and rotational thromboelastometry (ROTEM). The surgical procedure and bleeding management is explained further in an additional file (see Additional file 3).

### Statistical methods

Categorical data were presented as numbers and percentages. Continuous variables with skewness and kurtosis within the range of ± 1 were considered normally distributed and were presented as mean ± standard deviation while non-normally distributed variables were presented as median and interquartile range. Intergroup comparison between categorical variables was analyzed with Chi-Square tests when the expected cell count was more than 5, otherwise Fisher’s exact test was used for 2×2 tables and Fisher Freeman Halton’s exact test for contingency tables exceeding 2×2. Normally distributed continuous variables were analyzed with two sample T-tests, and non-normally distributed continuous variables were analyzed with Mann–Whitney U-tests. To ensure compatibility with the statistical analyses, lab values that were outside the measurable range were recoded to reflect the upper or lower limit value.

Uni- and multivariable analyses were performed using logistic regression. The multivariable analyses relied on complete case analysis. The full uni- and multivariable analysis is presented in an additional file (see Additional file 1). All variables in the multivariable analyses were examined for multicollinearity or correlation. The reliability of the multivariable model was assessed with the omnibus test and the Hosmer and Lemeshow test. Selection of variables to include in the multivariable model were determined using an a priori approach: age, hypertension, coronary artery disease, preoperative creatinine, any malperfusion, DeBakey type 1, and CPB time as they have all been shown to predict massive bleeding [5]. Bleeding-related routine lab variables (APTT, platelet count, and PT (INR)) were forced into the model as they are readily available for ATAAD patients, and their potential predictive value for massive bleeding could be clinically useful. Preoperative APTT, preoperative platelets, and preoperative creatinine were stratified into dichotomous variables. The cut-off points were generated using ROC curves and Youden’s index. Post hoc analyses were performed with the stratified variable replacing the continuous variable. Preoperative lactate, which differed between groups, was not part of the a priori variable set; in a post-hoc model it was not an independent predictor (p=0.33) and was therefore not retained. As a sensitivity analysis, the multivariable model was repeated with treatment era (three equal calendar periods) included. The multivariable model is explained further in an additional file (see Additional file 2).

A *p*-value <0.05 was considered statistically significant for all tests. All statistical analyses were performed on IBM SPSS Statistics for Mac, Version 29.0.0.0.

## Results

**Fig. 1:**
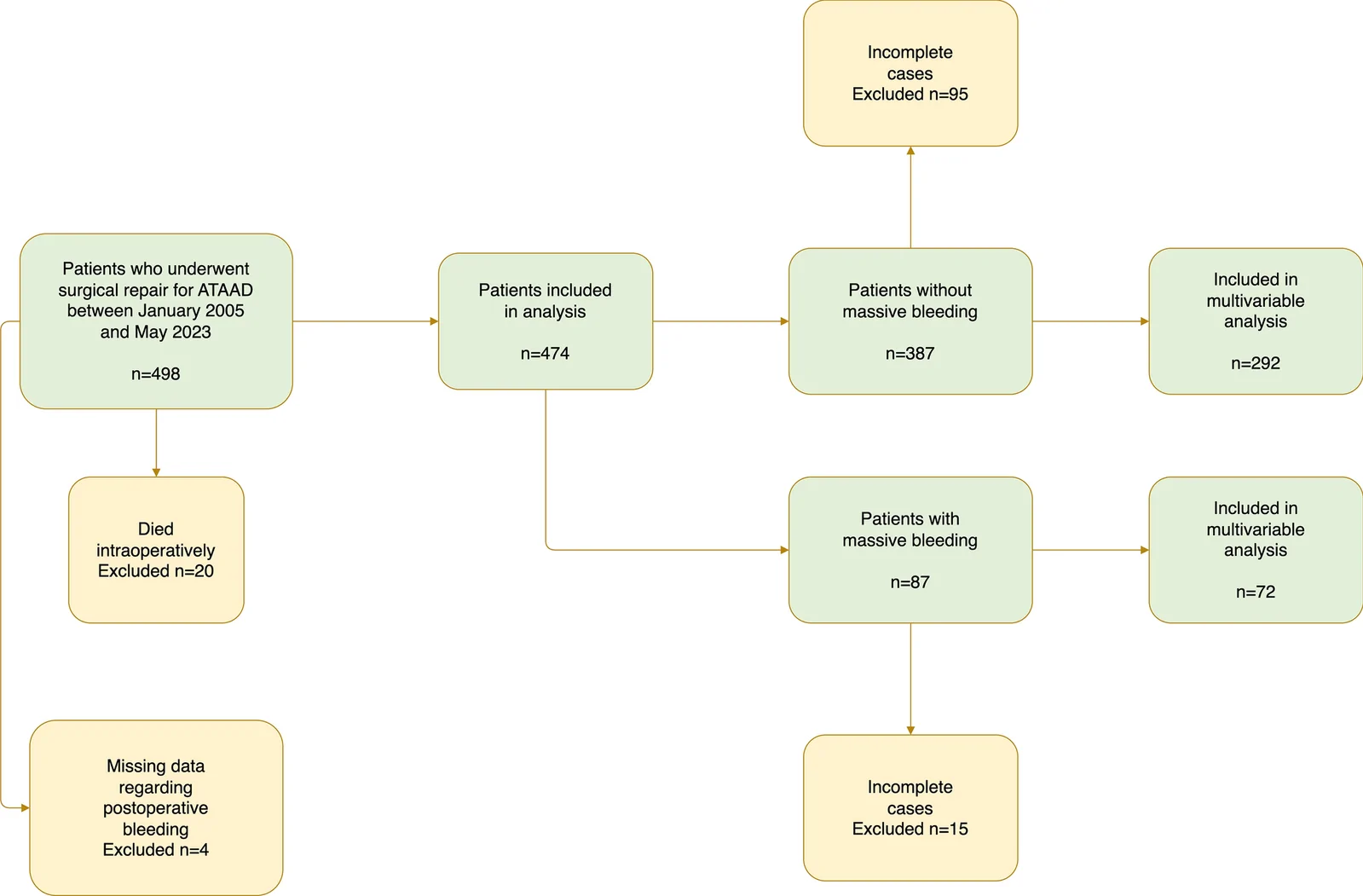
Flowchart of the inclusion and exclusion criteria

A total of 498 patients underwent aortic repair surgery for ATAAD between January 2005 and May 2023. Mors in tabula was 4.0% (n=20) during the index procedure, and 0.8% (n=4) patients had incomplete data and were excluded from further analysis. A total of 474 patients were included in the study, and 18.4% (n=87) of these suffered postoperative massive bleeding (Fig. 1). The distribution of the individual BART components among the 87 patients with massive bleeding is shown in an additional file (see Additional file 4): Transfusion of >10 U red blood cells within 24 h (n=55), reoperation for bleeding or tamponade within 24 h (n=42), chest-tube bleeding >1500 ml/8 h (n=11), and death from haemorrhage within 30 days (n=5); components frequently co-occurred.

Baseline characteristics of the study population are shown in Table 1. The mean age of the study population was 64.8 years (± 11.3), the median BMI was 26 kg/m^2^ [23.5–29.4], and 38% of the population were females. The patients in the massive bleeding group were significantly older (66.0 ± 9.7 vs 64.5 ± 11.6, *p*=0.04) and had lower BMI (24.8 kg/m^2^ [22.3–27.8] vs 26.3 kg/m^2^ [23.5–29.8], *p*=0.03) compared to the control group. They were also more likely to have diabetes mellitus (34.5% vs 16%, *p*<0.01) and hyperlipidemia (11.5% vs 5%, *p*=0.04). The patients with massive bleeding more often underwent previous cardiac surgery (4.7% vs 0.8%, *p*=0.02) and reported shorter symptom duration (time from onset of symptom until surgical incision) (6:06 [4:18–10:29] hh:mm vs 7:04 [4:53–15:04] hh:mm, *p*=0.04).

**Table 1:** Baseline characteristics of the study population

Baseline data	All (n=474)	Patients with massive bleeding (n=87)	Controls (n=387)	P-value	Missing
Age (years)^I^	64.8 ± 11.3	66.0 ± 9.7	64.5 ± 11.6	0.04*	0
BMI (kg/m^2^)^II^	26.0 [23.5–29.4]	24.8 [22.3–27.8]	26.3 [23.5–29.8]	0.03*	93 (19.6)
Women^III^	180 (38.0)	29 (33.3)	151 (39.0)	0.32	0
Hypertension^III^	263 (55.5)	56 (64.4)	207 (53.5)	0.07	0
Diabetes mellitus^III^	92 (19.4)	30 (34.5)	62 (16.0)	<0.01*	0
COPD^III^	25 (5.3)	8 (9.2)	17 (4.4)	0.07	0
History of smoking^III^	151 (33.6)	29 (33.7)	122 (33.5)	0.98	24 (5.1)
Hyperlipidemia^III^	21 (6.6)	9 (11.5)	12 (5.0)	0.04*	154 (32.5)
Coronary artery disease^III^	35 (7.4)	9 (10.3)	26 (6.7)	0.24	0
Known thoracic aneurysm^III^	55 (11.6)	13 (14.9)	42 (10.9)	0.29	1 (0.2)
Marfan syndrome^III^	27 (5.7)	5 (5,7)	22 (5.7)	0.98	0
Other connective tissue disease^IV^	5 (1.1)	0	5 (1,3)	0.59	0
Family history of dissection^IV^	22 (4.6)	2 (2.3)	20 (5.1)	0.40	0
Family history of aneurysm^IV^	11 (2.3)	1 (1.1)	10 (2.6)	0.70	0
Previous cardiac surgery^IV^	7 (1.5)	4 (4.7)	3 (0.8)	0.02*	1 (0.2)
Previous aortic surgery^III^	19 (4.0)	5 (5.7)	14 (3.6)	0.36	1 (0.2)
Symptom duration (hh:mm)	6:50 [4:46–14:16]	6:06 [4:18–10:29]	7:04 [4:53–15:04]	0.04*	169 (35.7)
Chronic kidney disease^IV^	5 (1.6)	2 (2.6)	3 (1.2)	0.60	154 (32.5)
Syncope^III^	83 (17.5)	19 (21.8)	64 (16.5)	0.24	0
Hypotensive shock^III^	113 (24.1)	27 (31.4)	86 (22.5)	0.08	6 (1.3)
Preoperative cardiac arrest^IV^	20 (4.2)	3 (3.4)	17 (4.4)	1.00	0
Sudden pain^III^	431 (91.1)	79 (90.8)	352 (91.2)	0.91	1 (0.2)
Cardiac tamponade^III^	80 (16.9)	19 (22.1)	61 (15.8)	0.16	1 (0.2)
Any malperfusion^III^	188 (39.7)	41 (47.1)	147 (38.0)	0.12	0
No malperfusion (M0)^III^	286 (60.3)	46 (52.9)	240 (62.0)	0.12	0
Coronary malperfusion (M1)^III^	52 (11.0)	7 (8.0)	45 (11.6)	0.35	2 (0.4)
Supra-aortic malperfusion (M2)^III^	95 (20.1)	22 (25.3)	73 (18.9)	0.19	1 (0.2)
Visceral, renal or lower-extremity malperfusion (M3)^III^	92 (19.4)	20 (23.0)	72 (18.6)	0.43	0
*Carotid dissection*^III^				0.86	19 (4.0)
None	234 (51.4)	40 (50.6)	194 (51.6)		
Unilateral	88 (19.3)	17 (21.5)	71 (18.9)		
Bilateral	133 (29.2)	22 (27.8)	110 (29.5)		
Intramural hematoma^III^	69 (14.6)	10 (11.5)	59 (15.3)	0.37	1 (0.2)
*DeBakey*^III^				0.45	2 (0.4)
Type 1	370 (78.4)	70 (81.4)	300 (77.7)		
Type 2	102 (21.6)	16 (18.6)	86 (22.3)		

*BMI* body mass index, *COPD* chronic obstructive pulmonary disease

Values are presented as numbers (%), mean ± standard deviation, median [interquartile range]

Times are presented as median [interquartile range] in the format hh:mm

^I^Two Sample T-test

^II^Mann–Whitney U Test

^III^Chi-square Test

^IV^Fisher’s Exact Test

P-value represents the comparison between patients with massive bleeding and patients without massive bleeding

p<0.05 is considered statistically significant and is marked with *

**Fig. 2:**
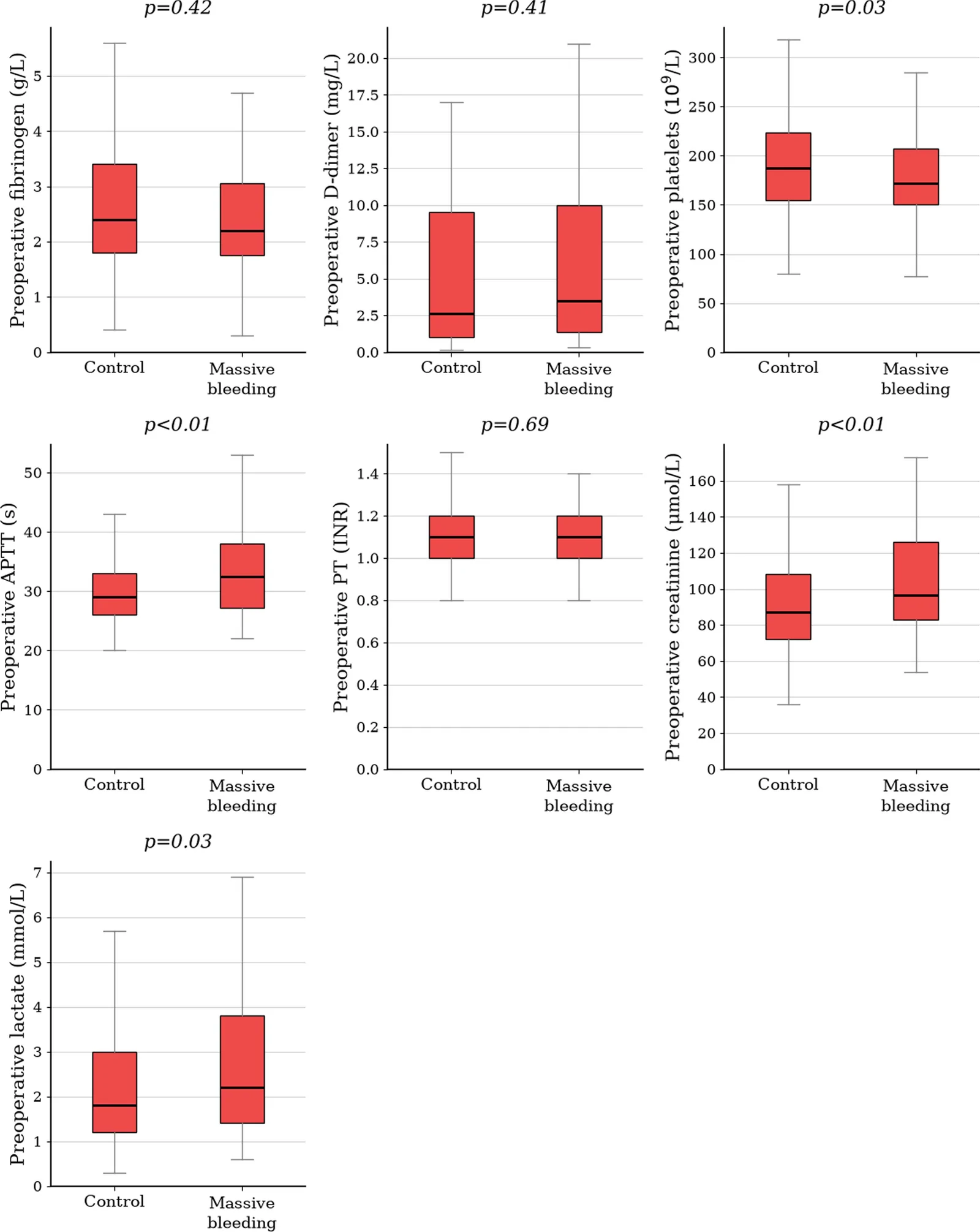
Box plot of preoperative laboratory results of the study population. Fig. 2: Preoperative fibrinogen, D-dimer, platelets, APTT, PT (INR), creatinine and lactate in the control group and in the massive bleeding group. Outliers are not visualized in the figures

Preoperative laboratory results of the study population are presented in Table 2 and visualized in box plots (Fig. 2). The patients in the massive bleeding group had significantly higher APTT (33 s [27–38] vs 29 s [26–33], *p*<0.01), creatinine (96.5 µmol/L [83.0–126.3] vs 87 µmol/L [72.0–108.0], *p*<0.01), and lactate (2.2 mmol/L [1.4–3.9] vs 1.8 mmol/L [1.2–3.0], *p*=0.03) and significantly lower platelet count (172 × 10^9^/L [150–211] vs 187 × 10^9^/L [155–224], *p*=0.03). The ROC analysis identified a cut-off of 34.5 s for APTT with an AUC of 0.624, a cut-off of 166.5 × 10^9^/L for platelets with an AUC of 0.579 and a cut-off of 82.5 for creatinine with an AUC of 0.628. In the massive bleeding group, 44.6% of patients had an APTT > 34.5 s (vs 21.6%, *p*<0.01), 47.5% had a platelet count < 166.5 × 10^9^/L (vs 33.4%, *p*=0.02), and 76.7% had a creatinine > 82.5 µmol/L (vs 56.7%, *p*<0.01). There were no significant differences in preoperative fibrinogen, D-dimer, or PT (INR).

**Table 2:** Preoperative laboratory results of the study population

Preoperative data	All (n=474)	Massive bleeding (n=87)	Control (n=387)	P-value	Missing
Fibrinogen (g/L)^II^	2.4 [1.8–3.3]	2.2 [1.7–3.1]	2.4 [1.8–3.4]	0.42	330 (69.6)
D dimer (mg/L)^II^	2.70 [1.10–10.00]	3.50 [1.29–10.00]	2.65 [1.00–9.60]	0.41	306 (64.6)
Platelets (10^9^/L)^II^	185 [155–222]	172 [150–211]	187 [155–224]	0.03*	59 (12.4)
APTT (s)^II^	29 [26–35]	33 [27–38]	29 [26–33]	<0.01*	95 (20.0)
PT (INR)^II^	1.1 [1.0–1.2]	1.1 [1.0–1.2]	1.1 [1.0–1.2]	0.69	64 (13.5)
Creatinine (µmol/L)^II^	89 [73.0–110.0]	97 [83.0–126.3]	87 [72.0–108.0]	<0.01*	14 (3.0)
Lactate (mmol/L)^II^	1.8 [1.2–3.2]	2.2 [1.4–3.9]	1.8 [1.2–3.0]	0.03*	12 (2.5)
APTT > 34.5^III^	99 (26.1)	33 (44.6)	66 (21.6)	<0.01*	95 (20.0)
Platelets < 166.5^III^	150 (36.1)	38 (47.5)	112 (33.4)	0.02*	59 (12.4)
Creatinine > 82.5^II^	278 (60.4)	66 (76.7)	212 (56.7)	<0.01*	14 (3.0)

Preoperative fibrinogen, D-dimer, platelets, APTT, PT (INR), creatinine and lactate in control group and in massive bleeding group

Values are presented as median [interquartile range]

^II^ Mann–Whitney U Test

^III^ Chi-square Test

P-value represents the comparison between patients with massive bleeding and patients without massive bleeding

p<0.05 is considered statistically significant and is marked with *

Patients with massive bleeding had longer CPB time (221 min [168–254] vs 170 min [134–206], *p*<0.01), longer cross-clamping time (93 min [65–144] vs 75 min [55–114], *p*<0.01), and longer HCA time (23 min [17–31] vs 20.0 min [15–25], *p*<0.01) compared to the control group (Table 3).

**Table 3:** Intraoperative data

Intraoperative data	All (n=474)	Patients with massive bleeding (n=87)	Control (n=387)	P-value	Missing
CPB time (min)^II^	174 [138–217]	221 [168–254]	170 [134–206]	<0.01*	0
Cross-clamping time (min)^II^	78 [58–119]	93 [65–144]	75 [55–114]	<0.01*	0
*HCA technique*^V^				0.16	6 (1.3)
Solely aortic cross clamp	24 (5.1)	3 (3.4)	21 (5.5)		
Solely circulatory arrest	245 (52.4)	42 (48.3)	203 (53.3)		
HCA + ACP	33 (7.1)	11 (12.6)	22 (5.8)		
HCA + RCP	166 (35.5)	31 (35.6)	135 (35.4)		
HCA time (min)^II^	20 [16–26]	23 [17–31]	20 [15–25]	<0.01*	33 (7.0)
HCA temperature (°C)^II^	19 [18–21]	18 [17–21]	19 [18–21]	0.07	3 (0.6)
*Arterial cannulation site*^V^				0.31	0
Femoral	337 (71.1)	68 (78.2)	269 (69.5)		
Axillary	13 (2.7)	3 (3.4)	10 (2.6)		
Direct aortic	115 (24.3)	15 (17.2)	100 (25.8)		
Ventricle	9 (1.9)	1 (1.1)	9 (2.3)		
*Distal surgical technique*^V^				0.38	0
Ascending	391 (82.5)	68 (78.2)	323 (83.5)		
Hemiarch	62 (13.1)	15 (17.2)	47 (12.1)		
Arch	21 (4.4)	4 (4.6)	17 (4.4)		
*Proximal surgical technique*^V^				0.19	0
Supracoronary graft + resuspension of aortic valve	318 (67.1)	57 (65.5)	261 (67.4)		
Supracoronary graft, no resuspension of aortic valve	31 (6.5)	2 (2.3)	29 (7.5)		
Mechanical Bentall	59 (12.4)	10 (11.5)	49 (12.7)		
Biological Bentall	38 (8.0)	10 (11.5)	28 (7.2)		
Ad modum David or Yacoub	7 (1.5)	2 (2.3)	5 (1.3)		
Aortic valve replacement + straight graft	21 (4.4)	6 (6.9)	15 (3.9)		

*CPB* cardiopulmonary bypass, *HCA* hypothermic circulatory arrest, *ACP* antegrade cerebral perfusion, *RCP* retrograde cerebral perfusion

Values are presented as numbers (%), median [interquartile range]

^II^Mann–Whitney U Test

^V^Fisher-Freeman-Halton Exact Test

P-value represents the comparison between massive bleeder group and non-massive bleeder group

p<0.05 is considered statistically significant and is marked with *

**Fig. 3:**
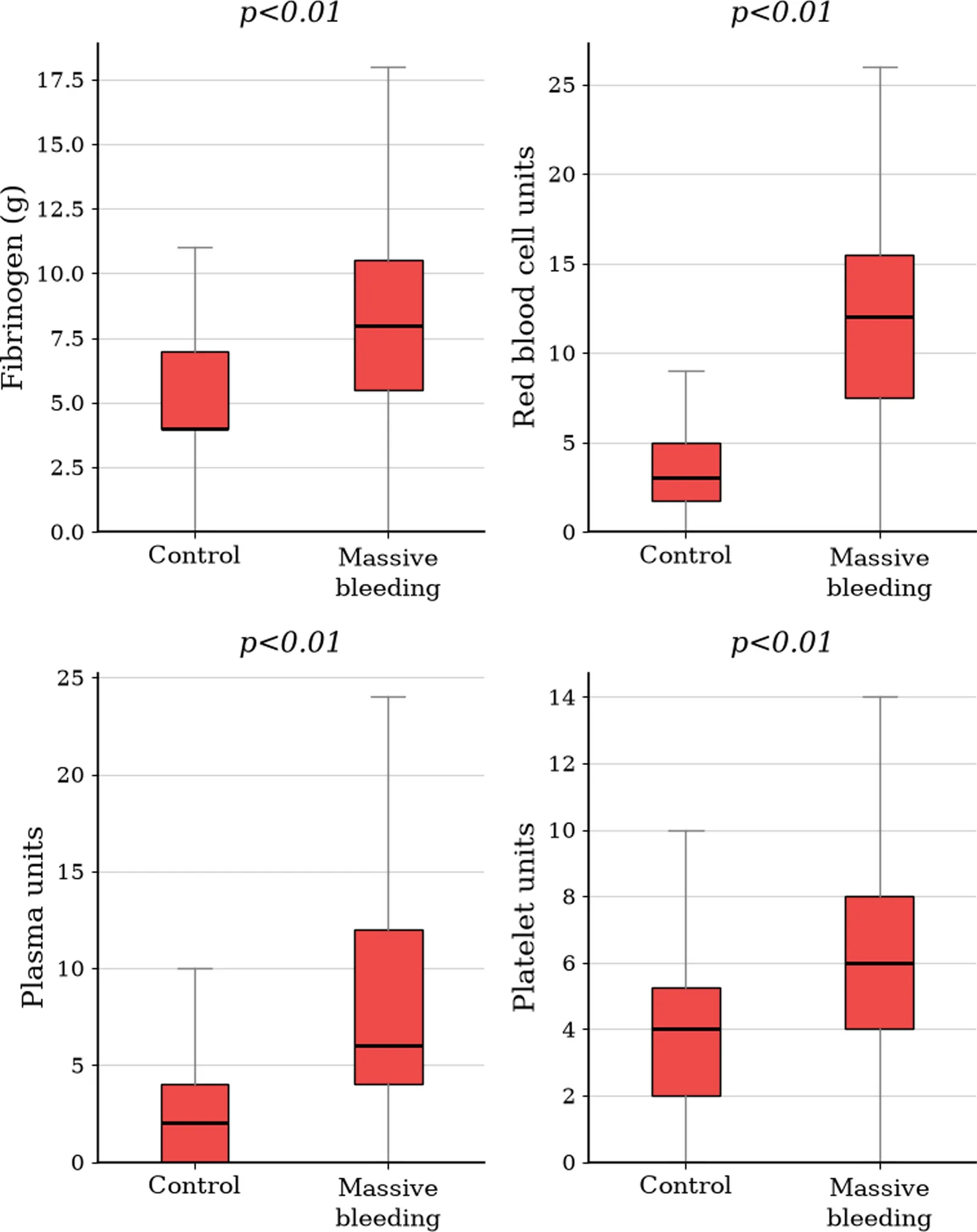
Box plot of postoperative substitutions. Fig. 3: Substitution of fibrinogen (g), red blood cell units, plasma units, and platelet units in the control group and in the massive bleeding group. Outliers are not visualized in the figures

Massive bleeding was associated with a significantly poorer outcome (Table 4) with a higher 30-day mortality (20.7% vs 5.7%,* p*=<0.01) as well as 90-day mortality (24.1% vs 6.5%, *p*=<0.01). The patients with massive bleeding received more transfusions of red blood cells (12U [7–16] vs 3U [1–5], p<0.01), platelets (6U [4–8] vs 4U [2–6], *p*<0.01), and fresh frozen plasma (6U [4–12] vs 2U [0–4], p<0.01) (Fig. 3). They also received more fibrinogen concentrate (8 g [5–11] vs 4 g [4–7], *p*<0.01), and more often received recombinant factor VII (57.4% vs 33.2%, *p*<0.01). Patients in the massive bleeding group also had significantly longer ICU-stay (7 days [5–10] vs 4 days [3–6], *p*<0.01) and a higher rate of postoperative complications including coma (16.1% vs 8.3%, *p*=0.03), multiple organ failure (8.9% vs 1.3%, *p*<0.01), tamponade (42.3% vs 2.1%, *p*<0.01), myocardial infarction (27.8% vs 8.9%, *p*=0.01), renal replacement therapy (21.8% vs 7.0%, *p*<0.01), deep sternal wound infection (6.4% vs 0.8%, *p*=0.01), atrial fibrillation (58.6% vs 43.8%, *p*=0.01), and ventilation exceeding 48 h (80.5% vs 26.9%, *p*<0.01).

**Table 4:** Postoperative data

Postoperative data	All (n=474)	Patients with massive bleeding (n=87)	Control (n=387)	P-value	Missing
90-day mortality^III^	46 (9.7)	21 (24.1)	25 (6.5)	<0.01*	2 (0.4)
30-day mortality^III^	40 (8.5)	18 (20.7)	22 (5.7)	<0.01*	4 (0.8)
Long term ventilation (> 48 h)^III^	174 (36.8)	70 (80.5)	104 (26.9)	<0.01*	1 (0.2)
Chest tube output during first 24 h (ml)^II^	640 [440–900]	1610 [790–2120]	600 [420–800]	<0.01*	136 (28.7)
Total chest tube output^II^	1000 [719–1603]	2105 [1385–3380]	935 [653–1350]	<0.01*	288 (60.8)
Reoperation due to bleeding^III^	58 (12.2)	51 (58.6)	7 (1.8)	<0.01*	0
Fibrinogen substitution (g)^II^	5 [8–4]	8 [11–5]	4 [7–4]	<0.01*	132 (27.8)
Recombinant Factor VII^II^	125 (36.5)	27 (57.4)	98 (33.2)	<0.01*	132 (27.8)
Red blood cell units^II^	4 [2–7]	12 [7–16]	3 [1–5]	<0.01*	3 (0.6)
Plasma units^II^	2 [0–6]	6 [4–12]	2 [0–4]	<001*	3 (0.6)
Platelet units^II^	4 [2–6]	6 [4–8]	4 [2–6]	<0.01*	3 (0.6)
Stroke^III^	97 (20.5)	22 (25.3)	75 (19.4)	0.22	0
Tamponade^III^	38 (11.9)	33 (42.3)	5 (2.1)	<0.01*	154 (32.5)
Coma^III^	46 (9.7)	15 (16.1)	32 (8.3)	0.03*	0
Atrial fibrillation^III^	220 (46.5)	51 (58.6)	169 (43.8)	0.01*	1 (0.2)
Myocardial infarction^III^	21 (10.7)	5 (27.8)	16 (8.9)	0.01*	277 (58.4)
Superficial sternal infection^IV^	12 (3.8)	2 (2.6)	10 (4.1)	0.73	154 (32.5)
Deep sternal infection^IV^	7 (2.2)	5 (6.4)	2 (0.8)	0.01*	154 (32.5)
Sepsis^IV^	1 (0.3)	0	1 (0.4)	0.02*	154 (32.5)
Multiple organ failure^IV^	9 (2.4)	5 (8.9)	4 (1.3)	<0.01*	100 (21.1)
Renal replacement therapy^IV^	46 (9.7)	19 (21.8)	27 (7.0)	<0.01*	0
Length of stay^II^	12 [8–20]	13 [9–22]	12 [7–20]	0.08	170 (35.9)
Length of stay in ICU^II^	4 [3–7]	7 [5–10]	4 [3–6]	<0.01*	260 (54.9)

Values are presented as numbers (%), median [interquartile range]

^II^Mann–Whitney U Test. ^III^Chi-square Test. ^IV^Fisher’s Exact Test

P-value represents the comparison between patients with massive bleeding and patients without massive bleeding

p<0.05 is considered statistically significant and is marked with *

In multivariable analysis, 23.2% (n=110) patients had missing data and were therefore excluded. The multivariable logistic regression revealed preoperative APTT per mmol increment (OR 1.04 (95% CI 1.01–1.07, *p*=0.01), preoperative APTT > 34.5 s (OR 2.95 (95% CI 1.60–5.47), *p*<0.01), preoperative creatinine > 82.5 µmol/L (OR 2.16 (95% CI 1.15–4.06), *p*=0.02), hypertension (OR 1.83 (95% CI 1.01–3.32), *p*<0.05) and CPB time per 10 min increment (OR 1.09 (95% CI 1.05–1.13, *p*<0.01)) as independent predictors of massive bleeding (Table 5).

**Table 5:** Multivariable logistic regression

Characteristics	Univariate analysis		Multivariable analysis	
	Odds Ratio (95% CI)	p-value	Odds Ratio (95% CI)	p-value
Age per year increment	1.01 (0.99–1.03)	0.28	1.03 (1.00–1.06)	0.06
Hypertension	1.57 (0.97–2.54)	0.07	1.83 (1.01–3.32)	<0.05*
Coronary artery disease	1.60 (0.72–3.55)	0.25	1.82 (0.62–5.30)	0.27
Preoperative creatinine per µmol increment	1.01 (1.00–1.01)	0.01*	1.00 (1.00–1.01)	0.13
Preoperative platelets per mmol increment	0.99 (0.99–1.00)	0.01*	1.00 (0.99–1.00)	0.06
Preoperative APTT per second increment	1.02 (1.00–1.04)	0.03*	1.04 (1.01–1.07)	0.01*
Preoperative PT (INR) per 1.0 increment	0.94 (0.50–1.78)	0.86	0.53 (0.22–1.27)	0.16
Any malperfusion	1.46 (0.91–2.32)	0.12	1.26 (0.71–2.24)	0.43
DeBakey type 1	1.25 (0.69–2.27)	0.46	0.55 (0.25–1.23)	0.15
CPB time (per 10 min increment)	1.10 (1.06–1.14)	<0.01*	1.09 (1.05–1.13)	<0.01*
Preoperative APTT > 34.5 s	2.92 (1.71–4.97)	<0.01*	2.95 (1.60–5.47)	<0.01*
Preoperative platelets > 166.5 × 10^9^/L	1.80 (1.10–2.95)	0.02*	1.59 (0.90–2.81)	0.11
Preoperative creatinine > 82.5 µmol/L	2.52 (1.47–4.33)	<0.01*	2.16 (1.15–4.06)	0.02*

*APTT* activated partial thromboplastin time, PT (INR) prothrombin complex (International Normalized Ratio), *CPB* cardiopulmonary bypass

Logistic regression, massive bleeding set as the dependent variable. A total of 364 complete cases was included in the analysis

For each of APTT, platelet count, and creatinine, the dichotomized variable was entered in a separate model in which it replaced its continuous counterpart; both estimates are presented together for readability

p<0.05 is considered statistically significant and is marked with *

## Discussion

In this study of patients undergoing surgical repair for ATAAD at our center, preoperative APTT and preoperative creatinine were independently associated with massive postoperative bleeding. In multivariable logistic regression, a preoperative APTT exceeding 34.5 s was the strongest predictor (OR 2.96). If confirmed in prospective studies, this readily available parameter may help identify patients who are at increased risk of massive bleeding and who could potentially benefit from earlier, more targeted hemostatic management. Furthermore, our study showed that the 18% of the patients who suffered postoperative massive bleeding had a significantly higher mortality rate and a higher rate of postoperative complications.

Preoperative APTT is a cost and time efficient coagulation assay that is accessible and commonly used to detect coagulation factor deficiencies and as a preoperative screening tool. It measures the time from activation of factor XII until clot formation and provides an indirect measurement of the intrinsic coagulation cascade pathway consisting of factor XII, factor XI, factor IX, and factor X [20]. Factor XII has previously been shown to decrease massively during moderate hypothermic cardiac arrest associated with ATAAD surgery. Factor XII deficiency also has been reported as an independent risk factor for reoperation due to bleeding, possibly as it requires more time to recover to preoperative levels than, for instance, factor VII [21]. In our study, we found that 44.6% of patients with massive bleeding had a preoperative APTT exceeding 34.5 s, compared to 21.6% of patients without massive bleeding.

As previously described, ATAAD poses a risk for consumption coagulopathy, adding to the hemostatic disturbance that naturally occurs during CPB [22–24]. Therefore, postoperative bleeding is common after surgical repair of ATAAD, and patients routinely require transfusion of blood components and administration of procoagulants perioperatively [25]. Reoperation for bleeding is associated with higher postoperative mortality both after surgery for ATAAD and after other cardiac surgeries [5, 26, 27]. ROTEM-guided protocols to avoid postoperative bleeding have a value in the bleeding management of patients undergoing aortic surgery but are limited in detecting the full complexity of the coagulopathy associated with ATAAD [28, 29]. Bleeding management protocols are generally based on analyses obtained after the cessation of CPB, delaying the administration of procoagulant drugs. Therefore, a simple preoperative screening tool may be valuable. Such a marker is not intended to replace intraoperative or post-CPB assessment but to complement it: APTT is available at admission in essentially all patients, and earlier awareness of bleeding risk carries little downside.

Notably, the pattern of biomarker abnormalities in our cohort does not fully correspond to that of an overt consumption coagulopathy. Only APTT and platelet count differed significantly between groups, whereas fibrinogen, D-dimer and PT (INR) did not. Several factors may explain this. D-dimer is near-universally elevated in acute aortic dissection and functions as a sensitive diagnostic marker [30, 31] and therefore has limited power to discriminate between bleeders and non-bleeders within a confirmed-dissection population. Fibrinogen, an acute-phase reactant, may remain normal or elevated early despite ongoing consumption. Both markers were also missing in a large proportion of patients, further limiting between-group comparison. Rather than reflecting overt disseminated intravascular coagulation, our findings may indicate that the degree of intrinsic-pathway derangement, captured by APTT, together with platelet consumption, discriminates the patients at highest risk of massive bleeding within an already coagulopathic population.

To avoid massive bleeding, an aggressive approach is needed, including transfusions and the administration of procoagulant drugs. In our study, patients with massive bleeding required significantly higher amounts of red blood cells, as well as fibrinogen, plasma, and platelets, and a larger proportion of the group required recombinant factor VII. The administration of procoagulant drugs is not without risk as an overuse of procoagulants may lead to thrombotic events such as stroke, which are also associated with higher postoperative mortality [32–34]. In our study, 20.5% of patients suffered postoperative stroke, with no significant difference between patients with massive bleeding and patients without massive bleeding. Both massive bleeding and overuse of procoagulants may cause major complications as well as mortality. This underscores the importance of identifying patients at increased risk for massive bleeding, as it could facilitate earlier and more aggressive procoagulant therapy for these patients.

In addition, our multivariable analysis revealed preoperative creatinine >82.5 µmol/L, time on CPB, and hypertension as independent predictors for massive bleeding. This is in line with previously published results [5, 35, 36].

A limitation of this study is that it only included patients who survived the initial surgical procedure, potentially excluding patients with extensive coagulopathy as they may have succumbed to the disease pre- or intraoperatively; the causes of the intraoperative deaths were not reliably recorded, precluding further characterization of this group. However, as the purpose of the study was to assess whether preoperative biomarkers of coagulation could be used to predict massive postoperative bleeding, its relevance only applies to patients who survive the surgical procedure. Furthermore, the study was limited by the inherent shortcomings of its retrospective design. Data were collected over an 18-year period, during which surgical technique and the capacity to manage coagulopathy evolved; the proportion of patients meeting criteria for massive bleeding increased significantly across the study period. In a sensitivity analysis adjusting for treatment era, preoperative APTT >34.5 s and creatinine >82.5 µmol/L remained independently associated with massive bleeding. Information on the timing and last dose of preoperative antiplatelet therapy was not consistently available, precluding assessment of its contribution to bleeding risk; however, preoperative APTT did not differ significantly between patients with and without coronary malperfusion (p>0.05), arguing against confounding of the APTT signal by this subgroup. The main strength of the study lies in its sizeable study population providing high statistical power.

The findings of this exploratory study are hypothesis-generating and prospective studies are required to validate these findings and determine whether they can be translated into clinical practice.

## Conclusion

In this retrospective cohort, a preoperative APTT exceeding 34.5 s was independently associated with postoperative massive bleeding after surgical repair for acute type A aortic dissection, as was a preoperative creatinine > 82.5 µmol/L. Massive bleeding was associated with significantly higher 30-day and 90-day mortality. These observations are hypothesis-generating and require prospective validation; if confirmed, preoperative APTT and creatinine may help identify patients at increased risk of massive postoperative bleeding.

## Supplementary Information

Supplementary Material 1.

Supplementary Material 2.

Supplementary Material 3.

Supplementary Material 4.

## Data Availability

All data generated or analysed during this study are included in this published article (and its supplementary information files).

## Abbreviations

ACP: Antegrade cerebral perfusion
ATAAD: Acute type A aortic dissection
APTT: Activated partial thromboplastin time
AUC: Area under curve
BART: Blood Conservation Using Antifibrinolytics in a Randomized Trial
BMI: Body mass index
COPD: Chronic obstructive pulmonary disease
CPB: Cardiopulmonary bypass
HCA: Hypothermic circulatory arrest
ICU: Intensive care unit
PT (INR): Prothrombin time (international standardized ratio)
RCP: Retrograde cerebral perfusion
ROC: Receiver operating characteristic

## References

[1] Melvinsdottir IH, Lund SH, Agnarsson BA, Sigvaldason K, Gudbjartsson T, Geirsson A. The incidence and mortality of acute thoracic aortic dissection: results from a whole nation study. Eur J Cardiothorac Surg. 2016;50(6):1111–7. 10.1093/ejcts/ezw235 27334108

[2] Howard DP, Banerjee A, Fairhead JF, Perkins J, Silver LE, Rothwell PM, et al. Population-based study of incidence and outcome of acute aortic dissection and premorbid risk factor control: 10-year results from the oxford vascular study. Circulation. 2013;127(20):2031–7. 10.1161/CIRCULATIONAHA.112.000483 PMC6016737 23599348

[3] Landenhed M, Engstrom G, Gottsater A, Caulfield MP, Hedblad B, Newton-Cheh C, et al. Risk profiles for aortic dissection and ruptured or surgically treated aneurysms: a prospective cohort study. J Am Heart Assoc. 2015;4(1):e001513. 10.1161/JAHA.114.001513 PMC4330075 25609416

[4] Czerny M, Schoenhoff F, Etz C, Englberger L, Khaladj N, Zierer A, et al. The impact of pre-operative malperfusion on outcome in acute type A aortic dissection: results from the GERAADA registry. J Am Coll Cardiol. 2015;65(24):2628–35. 10.1016/j.jacc.2015.04.030 26088302

[5] Zindovic I, Sjogren J, Bjursten H, Bjorklund E, Herou E, Ingemansson R, et al. Predictors and impact of massive bleeding in acute type A aortic dissection. Interact Cardiovasc Thorac Surg. 2017;24(4):498–505. 10.1093/icvts/ivw425 28093462

[6] Hansson EC, Geirsson A, Hjortdal V, Mennander A, Olsson C, Gunn J, et al. Preoperative dual antiplatelet therapy increases bleeding and transfusions but not mortality in acute aortic dissection type A repair. Eur J Cardiothorac Surg. 2019;56(1):182–8. 10.1093/ejcts/ezy469 30657880

[7] Scott J, Humphreys DR. Dissecting aortic aneurysm and disseminated intravascular coagulation. Br Med J. 1977;1(6052):24. 10.1136/bmj.1.6052.24 PMC1603633 831972

[8] Arima D, Suematsu Y, Yamada R, Matsumoto R, Kurahashi K, Nishi S, et al. Relationship of acute type A aortic dissection and disseminated intravascular coagulation. J Vasc Surg. 2022;75(5):1553-60 e1. 10.1016/j.jvs.2021.12.064 34998941

[9] Paparella D, Rotunno C, Guida P, Malvindi PG, Scrascia G, De Palo M, et al. Hemostasis alterations in patients with acute aortic dissection. Ann Thorac Surg. 2011;91(5):1364–9. 10.1016/j.athoracsur.2011.01.058 21396627

[10] Guan X, Li J, Gong M, Lan F, Zhang H. The hemostatic disturbance in patients with acute aortic dissection: a prospective observational study. Medicine. 2016;95(36):e4710. 10.1097/MD.0000000000004710 PMC5023888 27603366

[11] Guan XL, Wang XL, Liu YY, Lan F, Gong M, Li HY, et al. Changes in the hemostatic system of patients with acute aortic dissection undergoing aortic arch surgery. Ann Thorac Surg. 2016;101(3):945–51. 10.1016/j.athoracsur.2015.08.047 26603023

[12] Bartoszko J, Karkouti K. Managing the coagulopathy associated with cardiopulmonary bypass. J Thromb Haemost. 2021;19(3):617–32. 10.1111/jth.15195 33251719

[13] Van Poucke S, Stevens K, Marcus AE, Lance M. Hypothermia: effects on platelet function and hemostasis. Thromb J. 2014;12(1):31. 10.1186/s12959-014-0031-z PMC4265340 25506269

[14] Hofer J, Fries D, Solomon C, Velik-Salchner C, Ausserer J. A snapshot of coagulopathy after cardiopulmonary bypass. Clin Appl Thromb Hemost. 2016;22(6):505–11. 10.1177/1076029616651146 27268940

[15] Coakley M, Hall JE, Evans C, Duff E, Billing V, Yang L, et al. Assessment of thrombin generation measured before and after cardiopulmonary bypass surgery and its association with postoperative bleeding. J Thromb Haemost. 2011;9(2):282–92. 10.1111/j.1538-7836.2010.04146.x 21091865

[16] Zhou Y, Fan R, Jiang H, Liu R, Huang F, Chen X. A novel nomogram model to predict in-hospital mortality in patients with acute type A aortic dissection after surgery. J Cardiothorac Surg. 2024;19(1):362. 10.1186/s13019-024-02921-6 PMC11194955 38915077

[17] Fergusson DA, Hebert PC, Mazer CD, Fremes S, MacAdams C, Murkin JM, et al. A comparison of aprotinin and lysine analogues in high-risk cardiac surgery. N Engl J Med. 2008;358(22):2319–31. 10.1056/NEJMoa0802395 18480196

[18] Sievers HH, Rylski B, Czerny M, Baier ALM, Kreibich M, Siepe M, et al. Aortic dissection reconsidered: type, entry site, malperfusion classification adding clarity and enabling outcome prediction. Interact Cardiovasc Thorac Surg. 2020;30(3):451–7. 10.1093/icvts/ivz281 31755925

[19] Zindovic I, Sjogren J, Bjursten H, Ingemansson R, Ingimarsson J, Larsson M, et al. The coagulopathy of acute type A aortic dissection: a prospective, observational study. J Cardiothorac Vasc Anesth. 2019;33(10):2746–54. 10.1053/j.jvca.2019.02.013 30876766

[20] Ignjatovic V. Activated partial thromboplastin time. Methods Mol Biol. 2013;992:111–20. 10.1007/978-1-62703-339-8_8 23546708

[21] Li J, Guan X, Liu O, Wang X, Liu Y, Li H, et al. Changes in coagulation factor XII and its function during aortic arch surgery for acute aortic dissection-a prospective observational study. J Thorac Dis. 2018;10(7):4006–16. 10.21037/jtd.2018.06.127 PMC6105974 30174843

[22] Hansson EC, Dellborg M, Lepore V, Jeppsson A. Prevalence, indications and appropriateness of antiplatelet therapy in patients operated for acute aortic dissection: associations with bleeding complications and mortality. Heart. 2013;99(2):116–21. 10.1136/heartjnl-2012-302717 23048167

[23] ten Cate JW, Timmers H, Becker AE. Coagulopathy in ruptured or dissecting aortic aneurysms. Am J Med. 1975;59(2):171–6. 10.1016/0002-9343(75)90351-4 1155477

[24] Zhou C, Li Y, Yan Y, Feng D, Wei M, Wen J. Changes in coagulation and fibrinolysis systems during the perioperative period of acute type A aortic dissection. Heart Surg Forum. 2021;24(2):E223–30. 10.1532/hsf.3503 33798057

[25] Liu Y, Han L, Li J, Gong M, Zhang H, Guan X. Consumption coagulopathy in acute aortic dissection: principles of management. J Cardiothorac Surg. 2017;12(1):50. 10.1186/s13019-017-0613-5 PMC5468986 28606160

[26] Unsworth-White MJ, Herriot A, Valencia O, Poloniecki J, Smith EE, Murday AJ, et al. Resternotomy for bleeding after cardiac operation: a marker for increased morbidity and mortality. Ann Thorac Surg. 1995;59(3):664–7. 10.1016/0003-4975(94)00995-3 7887708

[27] Ranucci M, Bozzetti G, Ditta A, Cotza M, Carboni G, Ballotta A. Surgical reexploration after cardiac operations: why a worse outcome? Ann Thorac Surg. 2008;86(5):1557–62. 10.1016/j.athoracsur.2008.07.114 19049749

[28] Larsson M, Zindovic I, Sjogren J, Svensson PJ, Strandberg K, Nozohoor S. A prospective, controlled study on the utility of rotational thromboelastometry in surgery for acute type A aortic dissection. Sci Rep. 2022;12(1):18950. 10.1038/s41598-022-23701-z PMC9643344 36347972

[29] Karrar S, Reniers T, Filius A, Bunge JJH, Bekkers JA, Hoeks SE, et al. Rotational thromboelastometry-guided transfusion protocol to reduce allogeneic blood transfusion in proximal aortic surgery with deep hypothermic circulatory arrest. J Cardiothorac Vasc Anesth. 2022;36(4):1029–39. 10.1053/j.jvca.2021.08.020 34518103

[30] Nazerian P, Mueller C, Soeiro AM, Leidel BA, Salvadeo SAT, Giachino F, et al. Diagnostic accuracy of the aortic dissection detection risk score plus D-Dimer for Acute Aortic Syndromes: the ADvISED prospective multicenter study. Circulation. 2018;137(3):250–8. 10.1161/CIRCULATIONAHA.117.029457 29030346

[31] Suzuki T, Distante A, Zizza A, Trimarchi S, Villani M, Salerno Uriarte JA, et al. Diagnosis of acute aortic dissection by D-dimer: the International Registry of Acute Aortic Dissection Substudy on Biomarkers (IRAD-Bio) experience. Circulation. 2009;119(20):2702–7. 10.1161/CIRCULATIONAHA.108.833004 19433758

[32] Chemtob RA, Fuglsang S, Geirsson A, Ahlsson A, Olsson C, Gunn J, et al. Stroke in acute type A aortic dissection: the Nordic Consortium for ACUTE TYPE A Aortic Dissection (NORCAAD). Eur J Cardiothorac Surg. 2020;58(5):1027–34. 10.1093/ejcts/ezaa197 32688394

[33] Christoff AS, Winlaw DS, Curtin J, Barnes EH, Egan JR. Recombinant activated factor VII in neonatal cardiac surgery. Eur J Cardiothorac Surg. 2019;55(5):817–22. 10.1093/ejcts/ezy483 30715304

[34] Ponschab M, Landoni G, Biondi-Zoccai G, Bignami E, Frati E, Nicolotti D, et al. Recombinant activated factor VII increases stroke in cardiac surgery: a meta-analysis. J Cardiothorac Vasc Anesth. 2011;25(5):804–10. 10.1053/j.jvca.2011.03.004 21596585

[35] Ranucci M, Castelvecchio S, Menicanti L, Frigiola A, Pelissero G. Risk of assessing mortality risk in elective cardiac operations: age, creatinine, ejection fraction, and the law of parsimony. Circulation. 2009;119(24):3053–61. 10.1161/CIRCULATIONAHA.108.842393 19506110

[36] Wu ZN, Guan XL, Xu SJ, Wang XL, Li HY, Gong M, et al. Does preoperative serum creatinine affect the early surgical outcomes of acute Stanford type A aortic dissection? J Chin Med Assoc. 2020;83(3):266–71. 10.1097/JCMA.0000000000000264 PMC13048193 31990819

