# Supplementary material for: Preoperative levels of biomarkers of coagulation to predict massive bleeding after surgical repair for acute type A aortic dissection: a retrospective cohort study
Source: J Cardiothorac Surg. 2026 Oct 12;21:762. doi: 10.1186/s13019-026-04692-8 (PMC13660137; doi:10.1186/s13019-026-04692-8)
Supplement: Supplementary file 1 [file 13019_2026_4692_MOESM1_ESM.docx]

**Additional file 1: Full uni- and multivariable analysis of predictors of massive bleeding.**

|  | **Univariate analysis** |  | **Multivariable analysis** | | |  |  |  |  |  |  |  |
| --- | --- | --- | --- | --- | --- | --- | --- | --- | --- | --- | --- | --- |
|  | Odds Ratio (95% CI) | p-value | Odds Ratio (95% CI) | | | p-value |  |  |  |  |  |  |
| Age per year increment | 1.01 (0.99-1.03) | 0.28 | 1.03 (1.00-1.06) | | 0.06 | | |  |  |  |  |  |
| Women | 0.78 (0.47-1.28) | 0.32 |  | |  | | |  |  |  |  |  |
| BMI per kg/m^2^ | 0.94 (0.88-0.99) | 0.03* |  | |  | | |  |  |  |  |  |
| Hypertension | 1.57 (0.97-2.54) | 0.07 | 1.83 (1.01-3.32) | | <0.05* | | |  |  |  |  |  |
| Diabetes mellitus | 2.76 (1.64-4.64) | <0.01* |  | |  | | |  |  |  |  |  |
| COPD | 2.20 (0.92-5.29) | 0.08 |  | |  | | |  |  |  |  |  |
| History of smoking | 1.01 (0.6-1.66) | 0.97 |  | |  | | |  |  |  |  |  |
| Hyperlipidemia | 2.50 (1.01-6.18) | 0.05 |  | |  | | |  |  |  |  |  |
| Coronary artery disease | 1.60 (0.72-3.55) | 0.25 | 1.82 (0.62-5.30) | | 0.27 | | |  |  |  |  |  |
| Known thoracic aneurysm | 1.44 (0.74-2.81) | 0.29 |  | |  | | |  |  |  |  |  |
| Marfan syndrome | 1.01 (0.37-2.75) | 0.98 |  | |  | | |  |  |  |  |  |
| Other connective tissue disease | NA | NA |  | |  | | |  |  |  |  |  |
| Family history of dissection | 0.43 (0.10-1.88) | 0.26 |  | |  | | |  |  |  |  |  |
| Family history of aneurysm | 0.438 (0.06-3.47) | 0.44 |  | |  | | |  |  |  |  |  |
| Previous cardiac surgery | 6.24 (1.37-28.43) | 0.02* |  | |  | | |  |  |  |  |  |
| Previous aortic surgery | 1.62 (0.57-4.62) | 0.37 |  | |  | | |  |  |  |  |  |
| Preoperative creatinine per µmol increment | 1.01 (1.00-1.01) | 0.01* | 1.00 (1.00-1.01) | | 0.13 | | |  |  |  |  |  |
| Preoperative creatinine > 82.5 µmol/L | 2.52 (1.47-4.33) | <0.01* | 2.16 (1.15-4.06) | | 0.02* | | |  |  |  |  |  |
| Preoperative lactate per mmol increment | 1.12 (1.03-1.22) | <0.01* |  | |  | | |  |  |  |  |  |
| Preoperative D-dimer per mmol increment | 0.99 (0.94-1.04) | 0.75 |  | |  | | |  |  |  |  |  |
| Preoperative fibrinogen per mmol increment | 0.84 (0.58-1.22) | 0.36 |  | |  | | |  |  |  |  |  |
| Preoperative platelets per mmol increment | 0.99 (0.99-0.10) | 0.01* | 1.00 (0.99-1.00) | | 0.06 | | |  |  |  |  |  |
| Preoperative platelets > 166.5 x 10^9^/L | 1.80 (1.10-2.95) | 0.02* | 1.59 (0.90-2.81) | | 0.11 | | |  |  |  |  |  |
| Preoperative APTT per mmol increment | 1.02 (1.00-1.04) | 0.03* | 1.04 (1.01-1.07) | | 0.01* | | |  |  |  |  |  |
| Preoperative APTT > 34.5 s | 2.92 (1.71-4.97) | <0.01* | 2.95 (1.60-5.47) | | <0.01* | | |  |  |  |  |  |
| Preoperative PT (INR) per mmol increment | 0.94 (0.50-1.78) | 0.85 | 0.53 (0.22-1.27) | | 0.16 | | |  |  |  |  |  |
| Symptom duration (per hour increment) | 0.99 (0.97-1.00) | 0.11 |  | |  | | |  |  |  |  |  |
| Chronic kidney disease | 2.10 (0.34-12.78) | 0.42 |  | |  | | |  |  |  |  |  |
| Syncope | 1.41 (0.79-2.51) | 0.24 |  | |  | | |  |  |  |  |  |
| Hypotensive shock | 1.58 (0.94-2.64) | 0.08 |  | |  | | |  |  |  |  |  |
| Preoperative cardiac arrest | 0.78 (0.22-2.71) | 0.69 |  | |  | | |  |  |  |  |  |
| Sudden pain |  |  |  | |  | | |  |  |  |  |  |
| Cardiac tamponade | 1.52 (0.85-2.70) | 0.16 |  | |  | | |  |  |  |  |  |
| Any malperfusion | 1.46 (0.91-2.32) | 0.12 | 1.26 (0.71-2.24) | | 0.43 | | |  |  |  |  |  |
| No malperfusion (M0) | 0.69 (0.43-1.10) | 0.12 |  | |  | | |  |  |  |  |  |
| Coronary malperfusion (M1) | 0.66 (0.29-1.52) | 0.33 |  | |  | | |  |  |  |  |  |
| supra-aortic malperfusion (M2) | 1.21 (0.92-1.58) | 0.18 |  | |  | | |  |  |  |  |  |
| Visceral, renal or lower-extremity malperfusion (M3) | 1.09 (0.91-1.32) | 0.35 |  | |  | | |  |  |  |  |  |
| *Carotid dissection* |  |  |  | |  | | |  |  |  |  |  |
| None^Ref^ |  | 0.86 |  | |  | | |  |  |  |  |  |
| Unilateral | 1.16 (0.62-2.18) | 0.64 |  | |  | | |  |  |  |  |  |
| Bilateral | 0.96 (0.54-1.70) | 0.89 |  | |  | | |  |  |  |  |  |
| Intramural hematoma | 0.72 (0.35-1.47) | 0.37 |  | |  | | |  |  |  |  |  |
| DeBakey type 1 | 1.30 (0.72-2.35) | 0.39 | 0.55 (0.25-1.23) | | 0.15 | | |  |  |  |  |  |
| CPB time (per 10 min increment) | 1.10 (1.06-1.14) | <0.01* | 1.09 (1.05-1.13) | | <0.01* | | |  |  |  |  |  |
| Cross-clamping time (per min increment) | 1.01 (1.00-1.01) | <0.01* |  | |  | | |  |  |  |  |  |
| *HCA technique* |  |  |  | |  | | |  |  |  |  |  |
| Aortic cross clamp^Ref^ |  | 0.15 |  | |  | | |  |  |  |  |  |
| Solely circulatory arrest | 1.45 (0.41-5.08) | 0.56 |  | |  | | |  |  |  |  |  |
| HCA + ACP | 3.50 (0.86-14.33) | 0.08 |  | |  | | |  |  |  |  |  |
| HCA + RCP | 1.61 (0.46-1.61) | 0.46 |  | |  | | |  |  |  |  |  |
| HCA time (min) | 1.03 (1.01-1.05) | <0.01* |  | |  | | |  |  |  |  |  |
| HCA temperature (°C) | 0.95 (0.90-1.02) | 0.15 |  | |  | | |  |  |  |  |  |
| *Arterial cannulation site* |  |  |  | |  | | |  |  |  |  |  |
| Femoral^Ref^ |  | 0.34 |  | |  | | |  |  |  |  |  |
| Axillary | 1.19 (0.32-4.43) | 0.80 |  | |  | | |  |  |  |  |  |
| Direct aortic | 0.59 (0.32-1.09) | 0.09 |  | |  | | |  |  |  |  |  |
| Ventricle | 0.49 (0.06-4.02) | 0.51 |  | |  | | |  |  |  |  |  |
| *Distal surgical technique* |  |  |  | |  | | |  |  |  |  |  |
| Ascending^Ref^ |  | 0.44 |  | |  | | |  |  |  |  |  |
| Hemiarch | 1.52 (0.80-2.87) | 0.20 |  | |  | | |  |  |  |  |  |
| Arch | 1.12 (0.37-3.43) | 0.85 |  | |  | | |  |  |  |  |  |
| *Proximal surgical technique* |  |  |  | |  | | |  |  |  |  |  |
| Supracoronary graft + resuspension of aortic valve^Ref^ |  | 0.29 |  | |  | | |  |  |  |  |  |
| Supracoronary graft, no resuspension of aortic valve | 0.32 (0.07-1.36) | 0.12 |  | |  | | |  |  |  |  |  |
| Mechanical Bentall | 0.93 (0.45-1.96) | 0.86 |  | |  | | |  |  |  |  |  |
| Biological Bentall | 1.64 (0.76-3.56) | 0.22 |  | |  | | |  |  |  |  |  |
| Ad modum David or Yacoub | 1.83 (0.35-9.69) | 0.46 |  | |  | | |  |  |  |  |  |
| Aortic valve replacement + straight graft | 1.83 (0.68-4.93) | 0.23 |  | |  | | |  |  |  |  |  |
| Logistic regression, massive bleeding set as the dependent variable. A total of 364 complete cases was included in the multivariable analysis. For each of APTT, platelet count and creatinine, the dichotomized variable was entered in a separate model in which it replaced its continuous counterpart; both estimates are presented together for readability. Abbreviations: *COPD:* chronic obstructive pulmonary disorder, *APTT:* activated partial thromboplastin time, *PT:* prothrombin complex, *NA:* Not applicable, *CPB:* cardiopulmonary bypass, *HCA*: hypothermic circulatory arrest, *ACP*: antegrade cerebral perfusion, *RCP*: retrograde cerebral perfusion. *p*<0.05 is considered statistically significant and is marked with *. | | | | | | | |  |  |  |  |  |
|  | | |  |  | | | |  |  |  |  |  |
