# Supplementary material for: Preoperative levels of biomarkers of coagulation to predict massive bleeding after surgical repair for acute type A aortic dissection: a retrospective cohort study
Source: J Cardiothorac Surg. 2026 Oct 12;21:762. doi: 10.1186/s13019-026-04692-8 (PMC13660137; doi:10.1186/s13019-026-04692-8)
Supplement: Supplementary file 2 [file 13019_2026_4692_MOESM2_ESM.docx]

**Additional file 2: Multivariable model explained**

All variables in the multivariable analyses were analyzed for multicollinearity or correlation. Continuous variables were examined with linear regression, and a variance inflation factor (VIF) exceeding 3 was considered as indicative of multicollinearity. Correlation between categorical variables was examined using Phi-Coefficient, and correlation between continuous variables and categorical variables was examined with Point-Biserial correlation. Correlation coefficients less than ± 0.4 were considered insignificant and allowed the variables to be included in the model.

Age, symptom duration, hypertension, coronary artery disease, organ malperfusion, De Bakey type 1, preoperative dual antiplatelet therapy, preoperative creatinine, duration of CPB, and aortic cross clamp have previously been established as predictors of massive bleeding [5], and these variables were assessed for inclusion in the multivariable model. Symptom duration, preoperative dual antiplatelet therapy, preoperative D-dimer, and preoperative fibrinogen were excluded from the model due to excessive missing data. Given the inherent similarity of aortic cross-clamping time and CPB time, only CPB was included in the model.

Preoperative APTT, preoperative platelets, and preoperative creatinine were stratified into dichotomous variables with the use of post-hoc analyses. This was done after receiver operating characteristic curves (ROC-curves) were performed for each of these variables, and the best corresponding Youden’s indexes was obtained and set as cut-offs. For preoperative creatinine, 2 values rendered the same Youden’s index, and the value providing the highest sensitivity was chosen. The core model was run separately with each dichotomized variable replacing the original continuous variable. The odds ratios and the p-values for the dichotomized variables are presented together with the core model without adjusting the odds ratios and *p*-values from the core model.
