# Supplementary material for: Preoperative levels of biomarkers of coagulation to predict massive bleeding after surgical repair for acute type A aortic dissection: a retrospective cohort study
Source: J Cardiothorac Surg. 2026 Oct 12;21:762. doi: 10.1186/s13019-026-04692-8 (PMC13660137; doi:10.1186/s13019-026-04692-8)
Supplement: Supplementary file 3 [file 13019_2026_4692_MOESM3_ESM.docx]

**Additional file 3: Surgical procedure and bleeding management**

Arterial access was most frequently obtained through cannulation of the femoral artery or directly in the aorta (Seldinger wire-technique guided by transesophageal echocardiography) and less frequently in the axillary artery, or the left ventricle. In almost all cases, venous access was obtained through two-stage cannulation of the right atrium.

Deep hypothermic circulatory arrest (HCA) (<22°C) was used in almost all cases to allow distal repair with a few exceptions where the dissection was constrained to the most proximal ascending aorta and the repair could be done with aortic cross-clamping alone. In some HCA-cases, retrograde cerebral perfusion (RCP) or antegrade cerebral perfusion (ACP) was used as an additional neuroprotective technique alongside our standard protocol, which included topical cooling of the head, administration of thiopental (1 g), and administration of hydrocortisone (500 mg) before circulatory arrest. The distal surgical technique depended on the extent of the dissection and whether branches from the aortic arch were affected. Most patients had their graft anastomosed distally in the ascending aorta, while the remaining patients received either an hemiarch replacement or a total arch replacement. As soon as the distal anastomosis was completed, de-airing was performed with the patient in Trendelenburg-position, the vascular graft was clamped, and perfusion was restarted through a side branch of the vascular prosthesis. The proximal surgical technique depended on the location of the initial intimal tear and whether the root, the coronary vessels, and the aortic valve were affected. Most patients received a supracoronary graft in the ascending aorta, while a minority had a Bentall procedure, an isolated aortic valve replacement, or a valve sparing root replacement.

Decisions regarding details in bleeding management were at the discretion of each responsible surgeon. However, a standardized bleeding management protocol was routinely used, where coagulation was measured through blood analysis (B-haemoglobin, platelets, P-fibrinogen, P-PT (INR) and P-APTT) and rotational thromboelastometry (ROTEM). The ROTEM assays used were extrinsic thromboelastometry (Extem), fibrinogen thromboelastometry (Fibtem), and intrinsic thromboelastometry (Intem).

Four units of platelets, 2 g fibrinogen and 4 g tranexamic acid were routinely administered. Patients with B-hemoglobin below 90 g/L received red blood cell transfusions while platelets were transfused if maximum clot firmness (MCF) Extem did not reach 50 mm, MCF Fibtem exceeded 10 mm, or if platelet count dropped below 100 x 10^9^/L. Fibrinogen and/or plasma were administered if the MCF measured in Fibtem was less than 15 mm or if P-fibrinogen was less than 2 g/L. Plasma or prothrombin complex concentrate (PCC) was provided if the clotting time measured in Extem was longer than 100 seconds, the clotting time measured in Intem was longer than 240 seconds, the prothrombin time/international normalized ratio (P-PT (INR)) was more than 1.5, or if the partial thromboplastin time (P-APTT) exceeded 1.5 times the normal value.
