# Supplementary material for: Preoperative levels of biomarkers of coagulation to predict massive bleeding after surgical repair for acute type A aortic dissection: a retrospective cohort study
Source: J Cardiothorac Surg. 2026 Oct 12;21:762. doi: 10.1186/s13019-026-04692-8 (PMC13660137; doi:10.1186/s13019-026-04692-8)
Supplement: Supplementary file 4 [file 13019_2026_4692_MOESM4_ESM.docx]

**Additional file 4: Breakdown of the composite outcome**

| **BART component** | **n (of 87)** |
| --- | --- |
| Transfusion >10 U RBC within 24 h | 55 |
| Reoperation for bleeding / cardiac tamponade within 24 h | 42 |
| Chest-tube bleeding >1500 ml / 8 h | 11 |
| Death from haemorrhage within 30 days | 5 |

The overlap between criteria confirms that these are related, frequently co-occurring manifestations of the same haemorrhagic process rather than independent events.
